# Comparison of Different Drying Methods for Asparagus [*Asparagus cochinchinensis* (Lour.) Merr.] Root Volatile Compounds as Revealed Using Gas Chromatography Ion Mobility Spectrometry

**DOI:** 10.3389/fnut.2022.868209

**Published:** 2022-05-06

**Authors:** Gan-Lin Chen, Bo Lin, Feng-Jin Zheng, Wei-Hua Yu, Xiao-Chun Fang, Qian Shi, Yi-Feng Hu, Krishan K. Verma

**Affiliations:** ^1^Institute of Agro-Products Processing Science and Technology, Guangxi Academy of Agricultural Sciences, Nanning, China; ^2^Guangxi Key Laboratory of Fruits and Vegetables Storage-Processing Technology, Nanning, China; ^3^Institute of Biotechnology, Guangxi Academy of Agricultural Sciences, Nanning, China; ^4^Sugarcane Research Institute, Guangxi Academy of Agricultural Sciences, Nanning, China; ^5^Key Laboratory of Sugarcane Biotechnology and Genetic Improvement, Ministry of Agriculture and Rural Affairs, Nanning, China; ^6^Guangxi Key Laboratory of Sugarcane Genetic Improvement, Nanning, China

**Keywords:** *Asparagus cochinchinensis* (Lour.) Merr., drying, fingerprint spectrum, volatile compounds, HS-GC-IMS

## Abstract

Asparagus [*Asparagus cochinchinensis* (Lour.) Merr.] is a traditional herbal medicine plant commonly used to nourish yin, moisten dryness, and clear fire cough symptoms. Drying is an excellent option to conserve food materials, i.e., grains, fruits, vegetables, and herbs, reducing the raw materials volume and weight. This study aims to evaluate different drying approaches that could increase the value of asparagus, particularly as an ingredient in fast foods or as nutraceutical byproducts. The volatile components of asparagus roots were analyzed by using headspace-gas chromatography-ion mobility spectroscopy under different drying conditions, i.e., natural drying (ND) at ambient air temperature in the dark, well-ventilated room, temperature range 28–32°C, blast or oven drying at 50°C, heat pump or hot-air drying at temperature 50°C and air velocity at 1.5 ms^–1^ and vacuum freeze-drying at the temperature of −45°C and vacuum pressure of 10–30 Pa for 24 h. The findings revealed that the various drying processes had multiple effects on the color, odor index, and volatile compounds of the asparagus roots. As a result of the investigations, multiple characteristics of components, therefore, exploitation and comparison of various flavors; a total of 22 compounds were identified, such as alcohols, ketones, aldehydes, acids, esters, heterocyclic, and terpene. The present findings may help understand the flavor of the processed asparagus roots and find a better option for drying and processing.

## Introduction

The asparagus [*Asparagus cochinchinensis* (Lour.) Merr.] plant belongs to the family Liliaceae and is available at seashores, lightly wooded hillsides, roadsides, and unfertilized agricultural cultivated lands in China. It is a popular traditional herbal treatment for nourishing yin, moistening dryness, and clearing fire cough symptoms (1). Flavonoids and other phenolic substances found in asparagus have potent antioxidant properties (2, 3). The aroma of dried asparagus roots is determined by a group of volatile compounds (VCs), which is important for its quality. Drying is the key factor in processing dried asparagus roots, and the drying procedures might affect the VCs and flavors (1, 3, 4).

The efficiency of gas chromatography-ion mobility spectroscopy (GC-IMS) for the quantification and detection of volatile compounds in a variety of substrates has been demonstrated (4–8). In comparison to other analytical approaches, the IMS is characterized by a better sensitivity, minimal sample preparation, the benefits of rapidity, and relatively inexpensive analysis. The gas-phase ions separation in the IMS apparatus is based on their varying mobility inside a drift tube applied to a steady electric field maintained at atmospheric pressure. Due to the combination of the GC retention time and IMS drift time when employed as a GC detector, a two-dimensional separation of the volatile components in a given sample can be obtained. Recently, the potential for GC-IMS coupling to promote sensitivity and selectivity was explored (9, 10). Chemometric processing is required due to the large amount of data supplied by GC-IMS analyses. Headspace-gas chromatography combined with ion mobility spectrometry (HS-GC-IMS) has become an efficient approach used to quantify volatile compounds in solid or liquid samples with complex traits (11–13).

Drying is an ancient and unrivaled physical process driven by the energy of solar radiation for food-grains preservation for direct preparation of agro-food materials and further processing in the agro-industries and pharmaceutical products all year long (3, 14, 15). Nowadays, recent approaches have been thoroughly examined in terms of chemical and biological variations in the products subjected to the drying methods. It conserves the products and may significantly affect the quality of material (16, 17). The physical properties of the dried products are primarily fundamental in terms of rehydration, which is defined by the capacity of the dried food materials to back their natural flavors. Rehydration depends on various factors, i.e., before-treatment types, percentage of moisture, the technique of processing, and drying approaches (14, 15). Decreasing moisture content from the fresh material reduces bacterial development and multiplication and extends the shelf-life of the final product. Furthermore, the drying condition affects enzyme activities, sensory characteristics, and microbial development (3, 18, 19). The advanced drying procedures should get benefits like maximum efficiency of energy, product quality improvement, low cost, and reduced atmospheric losses (20).

The loss of energy during traditional hot air drying is quite substantial. As a result, various approaches have been developed to concentrate on restoring the exhausted air in the process (21). In this type of dryer, a refrigerator is used to recover the latent heat by water condensation. In this process, hot air is supplied to the product, releasing humid air. The air moves to the heat pump evaporator, where it is condensed, allowing the latent heat of vaporization to be reutilized to rewarm the drying air (14, 17, 22). The advantage of the heat pump drying strategy is that it reduces the time and temperature by lowering the relative humidity as compared to conventional hot-air dryers (17, 22). The blast or oven drying is one of the most employed approaches due to its low cost but implies exposure to oxygen and excess heat intensity (temperatures), which may affect the chemical composition of the food material. It creates a large vapor pressure differential between the center and the surface of the material, allowing rapid transport of moisture out of the product and preventing structural damages (23, 24). Freeze drying is the appropriate method for producing high-quality food/fruit material but has more expensive. This drying preserves sensory attributes, few authors documented that this might lead to loss of bioactive compounds (24–26).

Food drying causes sensory, physico-chemical, and nutritional changes (14). The morphological appearance of dried food products is very important for the initial assessment by the consumers. In the agro-food industries, color variations have enhanced morphological quality by applying coloring compounds, resulting in increased customers acceptability of foods (1, 3, 4, 15). Color variations caused by using various drying processes have already been demonstrated for a variety of plants (27–34). The stability of antioxidant compounds is regulated by several parameters, including the raw material, temperature, and processing time. The variation of phytochemical content may be affected by heat, time duration, oxygen levels, and availability of light (15, 35).

The volatile compounds have primarily been observed in aromatic herbs. Fresh and dried culinary herbs are the two most common ways to consume the plant. However, the fresh raw materials cannot be sold effectively worldwide. Significant volatile compounds are lost during the drying and preservation processes. As air temperature and wattage are enhanced, excess loss of volatile compounds occurs in convective hot air and blast drying processes (15, 21, 36, 37). The significant effect relative to other drying methods is the loss of volatile properties of the material (17, 22). The impact of dehydration on the volatile properties in various food materials implies that all drying processes dramatically reduce the total volatile content of fresh materials (38, 39).

In the present study, the volatile compounds from the dried asparagus roots were assessed by HS-GC-IMS under different drying conditions, such as ND (28–32°C), blast drying (BD, 50°C), heat-pump drying (HD, 50°C), and vacuum freeze drying (VFD, 45°C). The fingerprints spectrum of volatile compounds and principal component analysis (PCA) investigated the key flavor effects on volatile properties with various drying processes. To the best of our knowledge, the effect of different drying approaches on volatile compounds of asparagus roots has been studied limited. The asparagus root powder could be used in the future as a functional ingredient for the development of pharmaceutical and agro-industries.

## Materials and Methods

### Materials and Preparation of the Samples

Guidong no. 1 variety (G), an excellent variety of asparagus, was independently selected by the Institute of Biotechnology, Guangxi Academy of Agricultural Sciences (GxAAS), Nanning, Guangxi, China (22.49° N, 108.18° E). It is 2016 bred, using conventional breeding methods. The male and female parents were selected from the wild asparagus (root tubers) in Liuwanda Mountain, Yulin, Guangxi, China. The control (CK) was a farm variety in Guangxi common species (P). Three-year-old Guidong No. 1 and common species from the planting base of Chinese herbal medicines in Long’an, Guangxi, China, were used.

The asparagus plants root tubers of Guangxi common (P) and Guidong 1 (G) variety were collected from the planting base of the Chinese Herbal Medicine in Long’an, Guangxi, China, and Institute of Biotechnology, Guangxi Academy of Agricultural Sciences, Nanning, Guangxi, China. Dry asparagus roots were assessed for moisture content, which was nearly 3–7% (based on the drying curve and moisture content) and compared with different drying conditions. Analysis was performed to evaluate the color, odor index, volatile compounds, and fingerprints spectrum of the asparagus plant roots. The drying conditions were as follows: (a) ND, by spreading the asparagus roots on the net and drying at ambient air temperature in the dark, well-ventilated room, temperature range 28–32°C, (b) blast or oven drying (BD) at the temperature of 50°C (BD), (c) heat pump or hot-air drying at the temperature of 50°C and air velocity at 1.5 ms^–1^, and (d) VFD, pre-freeze at −18°C for 24 h, turn on the freezer, compressor work for 30 min, put the materials in the tray for the drying. The freeze-drying temperature was −45°C, and the vacuum pressure of 10–30 Pa for 24 h.

### Determination of Morphological Ultra-Structure of Asparagus Dried Roots by Scanning Electron Microscopy

Samples were fixed in a 1.5 ml (pentanediol) tipped centrifuge tube. The ratio of fixative and the samples was kept at 1:20 to ensure that the fixative was fully effective. In the fresh asparagus, roots were gently rinsed, the main vein was avoided, tissues about 3 mm × 7 mm in size were collected. The sample was quickly fixed in the pre-prepared fixative at room temperature in the dark to avoid light for more than 48 h. Samples were stored in a refrigerator (4°C) for further analysis (40). Dried roots samples were visualized using scanning electron microscopy (SEM) (Hitachi High-Tech Co., Ltd., Japan). SEM was carried out at 3.0 kV.

### Preparation of Asparagus Roots Samples and Quality Control

The dried asparagus root samples were frozen in liquid nitrogen and then ground into powder using an A11 basic Aika analytical grinder (Aika Instrument Equipment Co., Ltd., Guangzhou, China) and passed through a vibrating sieve with an aperture size of 0.30 mm. The prepared samples were stored in a refrigerator (−20°C) for further analysis.

### Analysis of Volatile Compounds Using Headspace-Gas Chromatography Combined With Ion Mobility Spectrometry

For HS-GC-IMS analysis, an Agilent Technologies 6890N gas chromatograph (Agilent, Waldbronn, Germany) coupled with automatic headspace sampler unit and a 2.5 ml syringe (Gerstel GmbH & Co. KG, Mühlheim, Germany) was equipped to an IMS module from G.A.S. (Gesellschaft fur Analytische Sensorsysteme mbH, Dortmund, Germany). 1.0 g root samples were placed in 20 mL headspace sampling bottles and incubated at 80°C (20 min). The centrifuge was set at 500 rpm, and the 200 μl sample was injected at 85°C. The gas chromatographic preparation was performed on a DB-FFAP (60 m × 0.25 mm, 1.0 μm film thickness, Agilent Technologies) capillary column (60°C), the analysis time duration was 30 min, the carrier gas was N_2_ (purity ≥99.999%), and the flow rate 0–2 min – 2 ml/min, 2–10 min-2–10 ml/min, 10–20 min-10–100 ml/min, and 20–30 min-100–150 ml/min. In the end, the IMS ionization chamber temperature was set at 45°C, the drift gas N_2_ (purity ≥99.999%), and the flow rate 150 ml/min. The GC-IMS flavor analyzer (FlavourSpec^®^, Shandong HaiNeng Scientific Instrument Co., Ltd., Shandong, China) was used.

Chromatographic peaks were tentatively identified based on the mass spectra and RI. Mass spectra of volatile compounds were compared with the National Institute of Standards and Technology (NIST) mass spectral database. Compounds were tentatively identified when the mass spectra similarity index was higher than 90%, and RI differed less than 5% from the available literature. Semi-quantitative analysis of relative contents of the volatile compounds was examined by area normalization.

### Determination of Physical Parameters

Plant samples were selected randomly and placed in the sample holder for measurement. Ten samples from each group were analyzed in parallel. The spectrophotometer (CM-3600A, Konica Minolta Investment Co., Ltd., China) examined the color of the dried roots under different drying conditions. Before use, the standard plate was used for calibration, and the *L* value (black and white-brightness), *a* value (red-green), and *b* value (yellow-blue) were recorded and expressed by the color difference value (ΔE), as the stated formula:


$$\Delta \,E=\sqrt{{\left(L-L_{0}\right)}^{2}+{\left(a-a_{0}\right)}^{2}+{\left(b-b_{0}\right)}^{2}}$$

Where L, a, and b are the color values and L_0_, a_0_, and b_0_ of the original sample values, respectively.

### Statistical Data Analysis

The aroma compound data was analyzed using Laboratory Analytical Viewer (LAV) and three plug-ins (Reporter, Gallery Plot, and Dynamic PCA plug-ins) as well as GC × IMS Library Search program from various perspectives using the built-in NIST software. The data were presented as mean ± standard deviation. ANOVA was carried out using SPSS 23.0 statistical program, and Duncan’s multiple comparison method was used to analyze the significance of differences. *p* < 0.05 indicates significant differences. The PCA diagram was created using Origin 2019b software.

## Results

### Gas-Phase Ion Mobility Spectra of Volatile Compounds in Asparagus Roots With Different Drying Conditions

**FIGURE 1:**
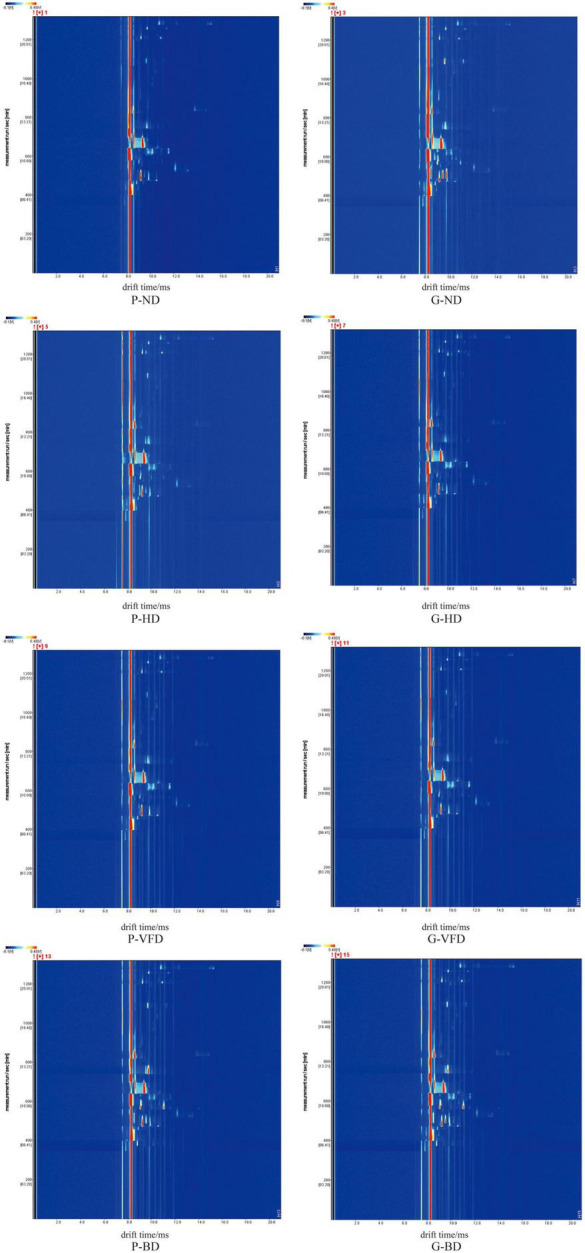
Two-dimensional overview of the gas phase-ion migration of volatile compounds in asparagus roots with different drying methods. P, common variety; G, Guidong no. 1 variety; ND, natural drying; HD, heat pump drying; VFD, vacuum freeze-drying; and BD, blast drying.

Gas chromatography-ion mobility spectroscopy examined volatile components variations in dried asparagus roots under different drying methods. The two-dimensional view of gas phase-ion movement was acquired. The reaction ion peak is the red vertical line on the left, and each point at the right side of the reactive ion peak represents volatile compounds. The color depth represents the content of volatile compounds, the white area represents the low compound content, and the red area represents high compound content (Figure 1).

**FIGURE 2:**
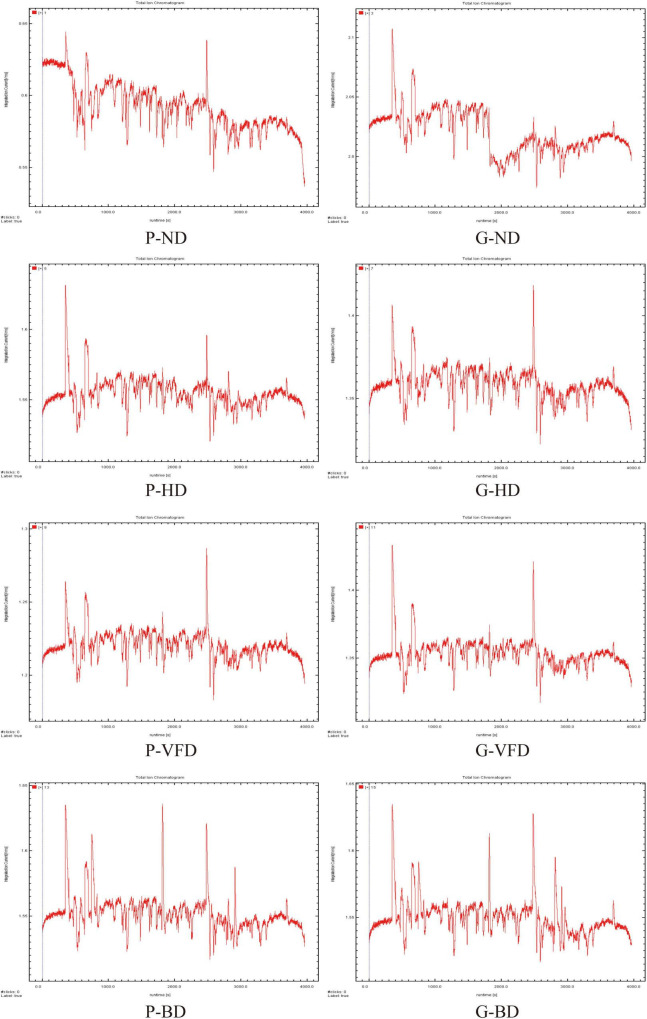
Chromatograms of volatile compounds extracted from dried asparagus roots using gas-chromatography ion mobility spectrometry. For compounds listed in Table 1.

As shown in Figure 1, most of the signals appear in the area where the drift time is 8.0–12.0 ms and the effective retention time (RI) was 400–1,000 s. The Retention Index (RI) and IMS databases quantified 22 compounds. The qualitative results are listed in Table 1, including the compound name, CAS number, RT, and keep time (s). The difference in volatile organic compounds of dried asparagus roots with different drying methods and varieties of asparagus is mainly manifested. For all 22 compounds, identification was based on chromatographic peak RI and the similarity index, which were higher than 90% (Figures 1, 2 and Table 1). Compared with the other drying methods, the red dot area becomes larger, and the color becomes darker, indicating that the heat treatment can keep the volatile compounds in the dried asparagus products and increase the content of some of them.

**TABLE 1:** Qualitative properties of volatile compounds in aspartame dried products.

Category	S. no.	Compound	CAS #	Formula	Retention time (RT)	Keep time(s)
Alcohols	1	Ethanol-D	64175	C_2_H_6_O	27.299	215
	2	Ethanol-M	64175	C_2_H_6_O	48.222	248
	3	1-Pentanol	71410	C_5_H_12_O	70.706	117
	4	3-hexen-1-ol	928961	C_6_H_12_O	54.781	128
Ketones	1	Acetone	67641	C_3_H_6_O	44.635	215
	2	2-octanone	111137	C_8_H_16_O	58.061	116
	3	Acetophenone	98862	C_8_H_8_O	51.947	57
	4	Cyclohexanone	108941	C_6_H_10_O	33.507	121
Aldehydes	1	(E,Z)-2,6-nonadienal	557482	C_9_H_14_O	64.773	72
	2	Pentanal	110623	C_5_H_10_O	18.379	248
	3	2-Methylbutanal	96173	C_5_H_10_O	79.693	103
	4	Benzaldehyde	100527	C_7_H_6_O	56.941	38
Acids	1	3-methylbutyric acid-M	503742	C_5_H_10_O_2_	107.123	88
	2	3-methylbutyric acid-D	503742	C_5_H_10_O_2_	69.322	55
	3	Acetic acid-D	64197	C_2_H_4_O_2_	34.378	122
	4	Acetic acid-M	64197	C_2_H_4_O_2_	29.517	211
Esters	1	Ethyl acetate-M	141786	C_4_H_8_O_2_	47.151	144
	2	Ethyl acetate-D	141786	C_4_H_8_O_2_	42.397	121
	3	1-Butanol	71363	C_4_H_10_O	79.490	99
Heterocyclic compound	1	2-methylpyrazine	109080	C_5_H_6_N_2_	52.660	145
	2	Tetramethylpyrazine	1124114	C_8_H_12_N_2_	85.993	148
Terpenes	1	Limonene	138863	C_10_H_16_	60.833	139

*M, monomer; D, dimer.*

### Qualitative Analysis of Asparagus Roots Volatile Compounds

Using ion migration drift time and RI to assess volatile compounds qualitatively, some of the volatile compounds in asparagus dried roots were found to have dimers and even trimers structures. Their RT is similar, but migration time is different. The qualitative analysis results of the volatile compounds of the samples are shown in Table 1. The volatile compound profiles of asparagus with varying drying approaches are the same, but compound content is other.

The result showed that a total of 22 volatile compounds were identified, such as 4 alcohols, 4 ketones, 4 aldehydes, 4 acids, 3 esters, 2 heterocyclic compounds, and 1 terpene (Table 1). Only a few asparagus root compounds were found in the forms of monomers and dimers, and the aromatic compounds in roots were dramatically altered during the process’s duration. The results showed differences in VCs of asparagus roots with different drying processes and marked the characteristic regions. Dimer and monomer signals were used to identify ethanol, acetic acid, ethyl acetate, and 3-methylbutyric acid at various keep times.

### Fingerprint Spectrum Analysis of Volatile Components in Asparagus Roots During Different Drying Methods

**FIGURE 3:**
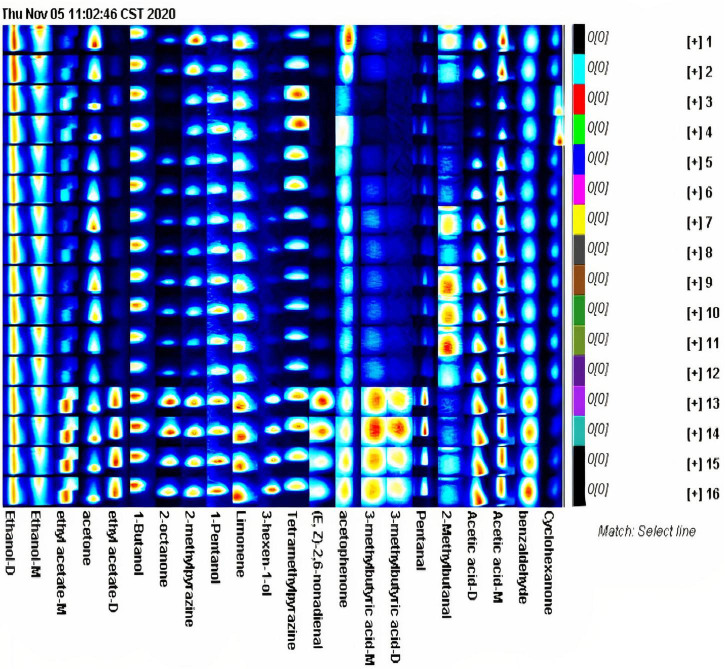
The Gallery Plot (fingerprint spectrum) of asparagus root samples with different drying methods.

According to the fingerprints of different processes generated by the Gallery Plot plug-in, the differences in volatile compounds between different processed samples can be compared qualitatively and quantitatively. Each row in the figure contains the volatile compounds contained in the samples, and each column corresponds to the same volatile compound between different samples. The color depth reflects the volatile compound content, and the brighter color represents higher content. The fingerprints may reveal the complete VCs information of each sample as well as VCs differences (Figure 3). In dried asparagus products, the major volatile compounds include ethanol, ethanol-monomer, ethyl acetate-monomer, acetone, ethyl acetate-dimer, 2-octanone, methylpyrazine, Limonene, tetramethylpyrazine, (E,Z)-2,6-nonadienal, valeraldehyde, acetic acid-dimer, acetic acid-monomer, and cyclohexanone (Figure 3). Although the content of the main volatile components of the processed samples by various drying processes is different, their components are retained. The content of some volatile compounds in the samples processed by hot-air drying is higher than natural drying. Ethyl acetate- monomer, ethyl acetate-dimer, isoamyl acetate, 2-octanone, methylpyrazine, (*trans*, *cis*)-2,6-nonadienal, isovaleric acid-monomer during hot-air drying, valeraldehyde, benzaldehyde, and cyclohexanone have higher content.

### Quality Differentiation of Asparagus Treated Roots With Different Drying Methods

**FIGURE 4:**
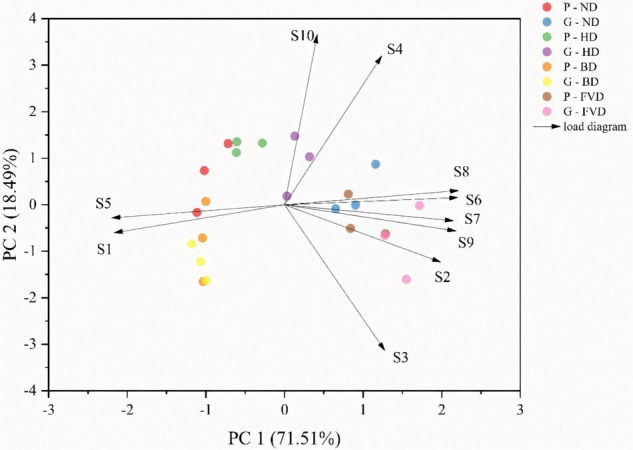
The analysis of principal component scores of different drying methods for the quality of asparagus samples.

Principal component analysis is a multivariate statistical method for analyzing the relationship between numerous variables (41). It may be used as a strong visualization tool, as well as a way to reduce data dimensionality and eliminate extraneous data (42, 43). PCA was performed on all samples using natural and alternative drying methods to understand the differences between the samples better. The samples were analyzed using PCA to get a rough idea of how their volatile makeup differed. The first two principal components explained about 90% of the variance between samples when the PCA was applied to the data represented in Table 2. Principal component 1 (PC1) accounted for 71.51% of the variance between the sample batches. Principal component 2 (PC2) was less significant, accounting for just 18.49% of the variation in the samples (Figure 4).

**TABLE 2:** The difference analysis of the relative intensity of each odor index of asparagus treated roots with different drying methods.

Array number	Sensor	Performance description	P-ND	G-ND	P-HD	G-HD	P-BD	G-BD	P-FVD	G-FVD
S1	W1C	Sensitive to aromatic compounds	0.9370 ± 0.01a	0.8112 ± 0.01d	0.8986 ± 0.02b	0.8594 ± 0.01c	0.9419 ± 0.00a	0.9433 ± 0.01a	0.8478 ± 0.02c	0.8065 ± 0.02d
S2	W5S	High sensitivity, very sensitive to nitrogen oxides	1.2689 ± 0.08d	3.4430 ± 0.16c	1.4406 ± 0.07d	1.6387 ± 0.04d	1.2618 ± 0.01d	1.2349 ± 0.03d	6.6845 ± 0.45b	7.9804 ± 0.53a
S3	W3C	Detection of aromatic components (especially ammonia)	1.0035 ± 0.01c	1.0583 ± 0.02b	1.0032 ± 0.01c	1.053 ± 0.04b	1.0327 ± 0.00*b*c	1.0543 ± 0.04b	1.0689 ± 0.02b	1.1158 ± 0.01a
S4	W6S	Used for selective detection of hydrogen (only detection of hydrogen in the aroma gas stream entering the electronic nose system)	1.0083 ± 0.01a	1.0063 ± 0.01a	1.0099 ± 0.00a	1.0130 ± 0.00a	0.9918 ± 0.00b	0.9909 ± 0.00b	1.0066 ± 0.00a	1.009 ± 0.00a
S5	W5C	Alkanes, aromatic compounds, compounds with little polarity	0.9813 ± 0.01a	0.9206 ± 0.01d	0.9590 ± 0.00b	0.9361 ± 0.00c	0.9743 ± 0.01a	0.9811 ± 0.00a	0.9277 ± 0.00*c*d	0.9072 ± 0.01e
S6	W1S	Mainly sensitive to methane in the environment, with high sensitivity	2.9834 ± 0.40e	8.0980 ± 0.54b	4.8442 ± 0.57d	7.14523 ± 0.55c	3.2764 ± 0.04e	3.2205 ± 0.33e	7.6751 ± 0.52*b*c	10.0492 ± 0.47a
S7	W1W	Mainly sensitive to sulfide (can detect 0.1 μg/g hydrogen sulfide) Very sensitive to many terpenes and organic sulfur compounds (mainly for the detection of odor, limonene, and piperazine)	3.7710 ± 0.75d	13.2188 ± 1.56a	5.3186 ± 0.42c	7.5072 ± 0.78b	3.7111 ± 0.10d	3.6863 ± 0.38d	14.5144 ± 0.58a	14.2113 ± 0.88a
S8	W2S	Ethanol detection is also sensitive to some aromatic compounds	1.4813 ± 0.11e	2.4005 ± 0.10b	1.7490 ± 0.10d	2.1871 ± 0.10c	1.4481 ± 0.01e	1.4502 ± 0.05e	2.3115 ± 0.09*b*c	2.6756 ± 0.06a
S9	W2W	Aromatic ingredients, sensitive to organic sulfur compounds	2.7881 ± 0.32*f*	10.8168 ± 0.66c	3.9123 ± 0.34e	5.5660 ± 0.38d	2.8094 ± 0.04*f*	2.8136 ± 0.27*f*	11.9450 ± 0.23b	13.0620 ± 0.22a
S10	W3S	Used to detect high-concentration alkanes (>100 μg/g)	1.0248 ± 0.03a	1.0362 ± 0.06a	1.0597 ± 0.02a	1.0484 ± 0.04a	1.0036 ± 0.07a	0.9608 ± 0.04a	1.0130 ± 0.03a	1.0047 ± 0.09a

*P, common variety; G, Guidong no. 1 variety; ND, natural drying; HD, heat pump drying; VFD, vacuum freeze-drying; and BD. blast drying. Significant variances in the values of the same group are indicated by different lowercase letters (n = 3, P < 0.05).*

### Comparison of the Asparagus Root Color With Different Drying Methods

The appearance of the dried asparagus roots varies depending on the drying processes. The color analysis characteristics indicating the L*, a*, b*, and ΔE values are mentioned in Table 3. The asparagus roots samples that underwent more drying processes were shrunken. However, the asparagus roots applied by the vacuum freeze-drying were white and with almost no shrinkage. The vacuum freeze-drying approach could balance the shape or structure of fresh asparagus root samples. Compared to fresh samples, the higher ΔE values were monitored in the VFD (both varieties). The lowest value of ΔE was observed in the natural drying (ND) process in both varieties. The highest L* values were observed in the VFD process compared to ND, HD, and BD processes (Table 3).

**TABLE 3:** Color analysis of asparagus treated with different drying methods.

Drying condition	L*	a*	b*	Δ E
P-initial	38.91 ± 1.19*bcd*	−1.06 ± 0.11c	1.56 ± 0.99d	38.96 ± 2.86*bc*
G-initial	41.03 ± 2.78*bc*	−1.39 ± 0.15c	0.84 ± 0.87d	41.07 ± 1.23b
P-ND	31.49 ± 4.11f	2.19 ± 1.56b	13.52 ± 4.20b	34.44 ± 5.42d
G-ND	34.28 ± 4.37*ef*	1.88 ± 0.89b	13.35 ± 3.8b	36.92 ± 5.23*cd*
P-HD	36.85 ± 3.79*cde*	3.69 ± 0.98a	18.61 ± 2.7a	41.49 ± 4.32b
G-HD	36.72 ± 4.59*de*	3.48 ± 1.26a	18.13 ± 3.64a	41.20 ± 5.15b
P-BD	37.33 ± 3.15*cde*	2.84 ± 0.99*ab*	18.11 ± 2.69a	17.60 ± 1.62e
G-BD	42.38 ± 3.69b	2.65 ± 1.91*ab*	20.12 ± 3.42a	20.53 ± 3.71e
P-FVD	82.66 ± 1.54a	−0.60 ± 0.32c	13.16 ± 1.73b	83.72 ± 1.41a
G-FVD	81.11 ± 7.98a	−1.20 ± 0.38c	10.21 ± 1.51c	84.17 ± 2.51a

*P, common variety; G, Guidong no. 1 variety; ND, natural drying; HD, heat pump drying; VFD, vacuum freeze-drying; and BD, blast drying. Significant differences are shown by different letters in the same column, while no significant differences are indicated by the same letters (P ≥ 0.05). n = 10.*

### Impact of Various Drying Processes on the Microstructure of Asparagus Dried Samples

**FIGURE 5:**
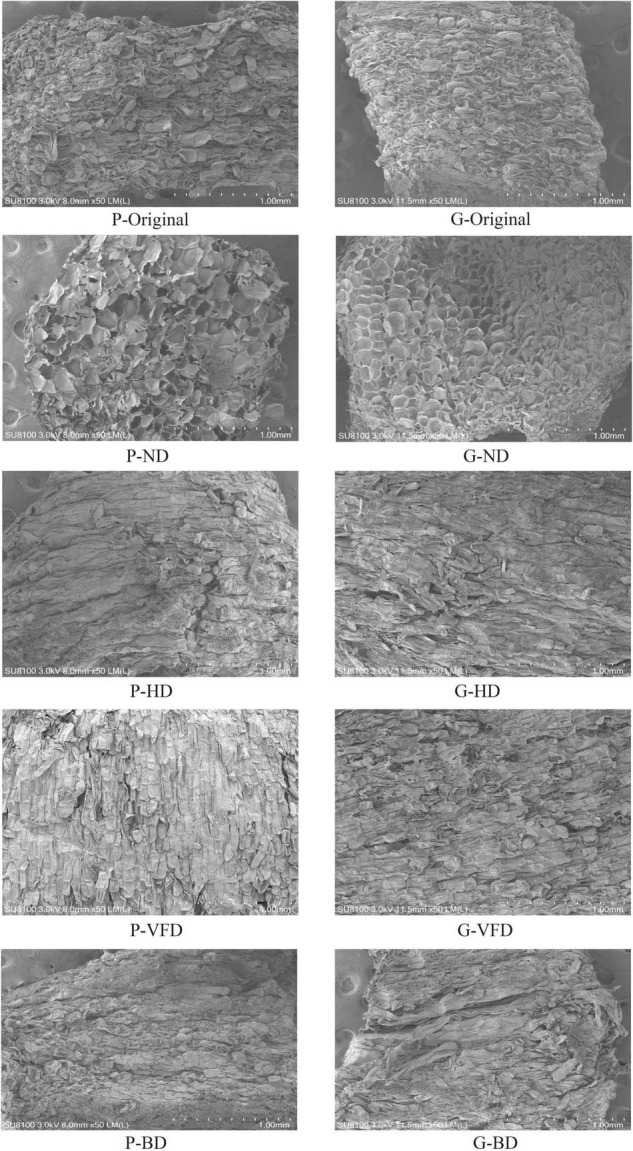
Ultrastructural changes of asparagus-dried products under different drying methods.

The influence of various drying processes on the morphological structure of fresh and dried asparagus roots was observed. It can be seen from Figure 5 that the cell structure of asparagus after drying and processing has changed dramatically as compared to the fresh sample. Fresh tissues of asparagus have tight epidermal cell structures, mostly rectangular, round, round-like, and rectangular-like, containing a large amount of mucus and water. After drying the mucus, the endothelial layer is prominent, and multiple layers of cells are superimposed. Due to the disappearance of the cell mass, the cell tissues change. Because of the different drying methods, the mucus quality and water distribution in the asparagus cells are other, and the binding force between the mucus quality and water changes, which leads to changes in the asparagus cell structure. Compared with fresh samples, the inside of the cell structure of the hot-air-dried (BD) and heat-pump-dried (HD) samples shrinks. After the mucus and water evaporate, a small part of the voids can be seen inside. The HD sample has more voids than the BD, but the structure remains tight.

## Discussion

A few additional value-adding processing approaches explored the health benefits of asparagus to increase its utilization and benefits to producers and consumers. Similar to other vegetables, asparagus changes color, chemical composition, and morphological texture as it dries (3, 15). When vegetables are dried with hot air, they usually shrink significantly and form dense structures (3). Moreover, the drying process affects enzymatic activities, sensory properties, and microbial growth (19). Food drying usually changes sensory as well as physico-chemical and nutritional qualities. It is important to utilize a suitable drying approach for each product and choose the right circumstances to minimize potential variations (44).

The visual appearance of dried food products plays a vital role in the first judgment made by the consumers, so maintaining the natural color is necessary. Browning processes, which enzymatic and non-enzymatic activities can generate, are the most common cause of color differences during drying (45). Long-duration drying and high temperatures cause color degradation of the original product. The variations in color seen under various drying conditions have already been established for various plant cultivars, such as pomegranate (30), soya (31), *Piper borbonense* (32), sour cherries (33), blueberry (28), black mulberry (27), and chokeberry (34).

Various parameters, including raw material, temperature, and process length, influence the stability of antioxidant compounds. During the drying process, the antioxidants are generally maintained. Furthermore, during the initial stage, there is a significant reduction of water, but the high vapor pressure produced by the evaporation of moisture reduces the partial pressure associated with the product and prevents the phenolic contents from oxidizing (46–48). To select the suitable drying process that results in high-quality dried products, it is essential to understand the retention of efficiency in antioxidants for each drying process (44). Conventionally, commercial asparagus powder is prepared by air-drying small bits of the asparagus spear and then crushing it (3, 49).

On the other hand, the drying process alters the volatile properties of asparagus powder. Some aromatic asparagus molecules get degraded during the procedure, while others, such as sulfur-containing molecules like dimethyl sulfide, might be generated during the drying process (50, 51). To minimize aroma depletion during storage, the production of such important volatiles should be controlled during the dehydration of asparagus plant samples (52–54). Advanced drying processes must be developed to acquire asparagus samples with stronger flavor characteristics. The selection of the drying process and the management of the drying process are among these approaches.

Based on the differences between asparagus root powder volatile properties, it is possible to infer that each drying process affects the composition of volatiles in various ways. Supposedly, the effects of drying on the volatile properties in root powders differ according to different major factors. The first factor was vapor pressure, which was enhanced by high vacuum in freeze-drying and by heat and air exchange during forced circulation of air over-drying; the second factor was VCs solubility in asparagus lipids and/or residual water, which is probably the major factor contributing to volatiles retention (3, 15, 24, 55). When identifying the relative contents of volatiles compounds, care should be taken through ion mobility spectrometry. In addition to the original contents of compounds in the sample, various factors could affect the peak areas of compounds. The highest peak area of a given volatile compound using HS-GC-IMS potentially indicates high content in the sample. Furthermore, drying circumstances, such as inlet temperature and volatility, impact spray drying retention. Increasing inlet temperature can improve flavor retention by reducing the consistent time duration (56, 57). As a result, carrier selection and drying processes affect the volatile compounds of spray-dried materials similar to King (58) and Feng et al. (41).

The odors originally contained in the asparagus, as well as the aromas produced through processing, may have a more significant impact on the overall flavor and taste of the product (59). Significant amounts of volatile chemicals are lost during the drying process. Few authors have stated that convective hot air drying and microwave drying experienced the maximum loss in VCs (21, 36, 60). The influence of dehydration on the volatile compounds in various food materials, such as oyster mushrooms, shitake mushrooms, and chanterelle mushrooms, claiming that different drying techniques severely reduced the total concentration of volatiles (38, 39, 61). The studies demonstrate that, compared to traditional or modern procedures performed as a specific treatment, a combination of approaches prefer to maintain a significant quantity of volatile properties, increasing the efficiency of some dried food materials and decreasing processing costs (62, 63).

## Conclusion

The food industries reinforce the investigation of key attributes and processing processes. Nowadays, producers have developed alternative and richer, fully prepared food materials with extended longer storage, and desiccated products fulfill all requirements. Furthermore, their operational and qualitative features should be as close to fresh plant materials as possible. In this aspect, the dryness of agricultural goods appears to be crucial in ensuring physical, chemical, and sensory qualities of the final products. Current research on the evaporation of agricultural goods using the heat-pump drying method has revealed that combination drying is the more effective approach for ensuring good quality while utilizing the least amount of energy. However, further research is needed to incorporate the kinds of treatments required to increase the texture, volatile properties, health-promoting characteristics, and attractive, sensory properties of novel drying procedures and their combination. However, each drying approach could be more suitable for various products depending on the targeted compounds. This could offer better opportunities for pharmaceutical and agro-industries in terms of product preservation and development. Afterward, the effects of different drying processes on the VCs of asparagus roots could be further analyzed based on factors, such as sensory evaluation, process differences, and varieties.

## Data Availability Statement

The original contributions presented in the study are included in the article/supplementary material, further inquiries can be directed to the corresponding author/s.

## Author Contributions

G-LC, BL, and F-JZ contributed to the conceptualization, methodology, data processing, investigation, resources, software, writing and editing the review, project administration, and funding acquisition. W-HY, X-CF, QS, Y-FH, and KV contributed to the resources, software, and data processing. All authors read and approved the article for publication.

## Conflict of Interest

The authors declare that the research was conducted in the absence of any commercial or financial relationships that could be construed as a potential conflict of interest.

## Publisher’s Note

All claims expressed in this article are solely those of the authors and do not necessarily represent those of their affiliated organizations, or those of the publisher, the editors and the reviewers. Any product that may be evaluated in this article, or claim that may be made by its manufacturer, is not guaranteed or endorsed by the publisher.

## References

[1] Yin Z Zhang J Kang W. Volatile components of *Asparagus cochinchinensis* stems. *Chem Nat Comp.* (2016) 52:1116–7. 10.1007/s10600-016-1879-x

[2] Makris DP Rossiter JT. Domestic processing of onion bulbs (Allium cepa) andasparagus spears (*Asparagus officinalis*): effect of flavonol content and antioxidant status. *J Agric Food Chem.* (2001) 49:3216–22. 10.1021/jf001497z 11453754

[3] Nindo CI Sun T Wang SW Tang J Powers JR. Evaluation of drying technologies for retention of physical quality andantioxid ants in asparagus (*Asparagus officinalis* L.). *Lebensm Wiss Technol.* (2003) 36:507–16. 10.1016/s0023-6438(03)00046-x

[4] Siccama JW Pegiou E Zhang L Mumm R Hall RD Boom RM Maltodextrin improves physical properties and volatile compound retention of spray-dried asparagus concentrate. *LWT Food Sci Technol.* (2021) 142:111058. 10.1016/j.lwt.2021.111058

[5] Kolb B Ettre LS. *Static Headspace-Gas Chromatography: Theory and Practice.* London: Wiley (2006).

[6] Karpas Z. Applications of ion mobility spectrometry (IMS) in the field of foodomics. *Food Res Int.* (2013) 54:1146–51. 10.1016/j.foodres.2012.11.029

[7] Wan Q Zhang L. Determination of acrylonitrile in solid waste by automatic headspace gas chromatography. *Proc Environ Sci.* (2016) 31:241–6. 10.1016/j.proenv.2016.02.032

[8] Khodadadi M Pourfarzam M. A review of strategies for untargeted urinary metabolomic analysis using gas chromatography mass spectrometry. *Metabolomics.* (2020) 16:66. 10.1007/s11306-020-01687-x 32419109

[9] Xie WQ Gong YX Yu KX. Determination of total sugar content in lignocellulosic hydrolysates by using a reaction headspace gas chromatographic technique. *Cellulose.* (2017) 24:4591–7. 10.1007/s10570-017-1483-7

[10] Vautz W Franzke J Zampolli S Elmi I Liedtke S. On the potential of ion mobility spectrometry coupled to GC pre-separation – A tutorial. *Anal Chim Acta.* (2018) 1024:52–64. 10.1016/j.aca.2018.02.052 29776547

[11] Xie WQ Chai XS. Rapid determination of moisture content in paper materials by multiple headspace extraction gas chromatography. *J Chromatogr A.* (2016) 1443:62–5. 10.1016/j.chroma.2016.03.059 27033986

[12] Hamilton SE Rossington MD Bertrand A. Development of an automated headspace gas chromatography instrument for the determination of residual solvents in pharmaceutical compounds and reaction mixtures. *Org Process Res Dev.* (2016) 20:189–94. 10.1021/acs.oprd.5b00367

[13] Arroyo-Manzanares N Garcia-Nicolas M Castell A Campillo N Vina P Lopez-Garcia I Untargeted headspace gas chromatography – Ion mobility spectrometry analysis for detection of adulterated honey. *Talanta.* (2019) 205:120123. 10.1016/j.talanta.2019.120123 31450393

[14] Calín-Sánchez A Lipan L Cano-Lamadrid M Kharaghani A Masztalerz K Carbonell-Barrachina A Comparison of traditional and novel drying techniques and its effect on quality of fruits, vegetables and aromatic herbs. *Foods.* (2020) 9:1261. 10.3390/foods9091261 PMC7554907 32916839

[15] Xia J Guo Z Fang S Gu J Liang X. Effect of drying methods on volatile compounds of burdock (*Arctium lappa* L.) root tea as revealed by gas chromatography mass spectrometry-based metabolomics. *Foods.* (2021) 10:868. 10.3390/foods.10040868 PMC8071549 33921154

[16] Szychowski PJ Lech K Sendra E Hernández F Figiel A Wojdyło A Kinetics, biocompounds, antioxidant activity, and sensory attributes of quinces as affected by drying method. *Food Chem.* (2018) 255:157–64. 10.1016/j.foodchem.2018.02.075 29571462

[17] Rahman MS. *Handbook of Food Preservation.* Colchester: Informa UK Limited (2020).

[18] Pydi-Setty Y Ramana-Murthy JV. Development of a model for drying of solids in a continuous fluidized bed dryer. *Ind J Chem Technol.* (2003) 10:477–82.

[19] Ozbek B Dadali G. Thin-layer drying characteristics and modelling of mint leaves undergoing microwave treatment. *J Food Eng.* (2007) 83:541–9. 10.1016/j.jfoodeng.2007.04.004

[20] Nemzer B Vargas L Xia X Sintara M Feng H. Phytochemical and physical properties of blueberries, tart cherries, strawberries, and cranberries as affected by different drying methods. *Food Chem.* (2018) 262:242–50. 10.1016/j.foodchem.2018.04.047 29751916

[21] Calín-Sánchez A Lech K Szumny A Figiel A Carbonell-Barrachina A. Volatile composition of sweet basil essential oil (*Ocimum basilicum* L.) as affected by drying method. *Food Res Int.* (2012) 48:217–25. 10.1016/j.foodres.2012.03.015

[22] Moses JA Norton T Alagusundaram K Tiwari B. Novel drying techniques for the food industry. *Food Eng Rev.* (2014) 6:43–55. 10.1007/s12393-014-9078-7

[23] Wojdyło A Figiel A Oszmianski J. Effect of drying methods with the application of vacuum microwaves on the bioactive compounds, color, and antioxidant activity of strawberry fruits. *J Agric Food Chem.* (2009) 57:1337–43. 10.1021/jf802507j 19170638

[24] Nunes JC Lago MG Castelo-Branco VN Oliveira FR Torres AG Perrone D Effect of drying method on volatile compounds, phenolic profile and antioxidant capacity of guava powders. *Food Chem.* (2016) 197:881–90. 10.1016/j.foodchem.2015.11.050 26617030

[25] Wojdyło A Figiel A Lech K Nowicka P Oszmianski J. Effect of convective and vacuum–microwave drying on the bioactive compounds, color, and antioxidant capacity of sour cherries. *Food Bioprocess Technol.* (2014) 7:829–41. 10.1007/s11947-013-1130-8

[26] Aydin E Gocmen D. The influences of drying method and metabisulfite pre-treatment on the color, functional properties and phenolic acids contents and bioaccessibility of pumpkin flour. *LWT Food Sci Technol.* (2015) 60:385–92. 10.1016/j.lwt.2014.08.025

[27] Chen Q Li Z Bi J Zhou L Yi J Wu X. Effect of hybrid drying methods on physicochemical, nutritional and antioxidant properties of dried black mulberry. *LWT Food Sci Technol.* (2017) 80:178–84. 10.1016/j.lwt.2017.02.017

[28] Zielinska D Michalska-Ciechanowska A. Microwave-assisted drying of blueberry (*Vaccinium corymbosum* L.) fruits: drying kinetics, polyphenols, anthocyanins, antioxidant capacity, colour and texture. *Food Chem.* (2016) 212:671–80. 10.1016/j.foodchem.2016.06.003 27374583

[29] Michalska-Ciechanowska A Wojdyło A Lech K Łysiak G Figiel A. Effect of different drying techniques on physical properties, total polyphenols and antioxidant capacity of blackcurrant pomace powders. *LWT.* (2017) 78:114–21. 10.1016/j.lwt.2016.12.008

[30] Tontul I Topuz A. Effects of different drying methods on the physicochemical properties of pomegranate leather (pestil). *LWT.* (2017) 80:294–303. 10.1016/j.lwt.2017.02.035

[31] Muliterno MM Rodrigues D De Lima FS Ida EI Kurozawa LE. Conversion/degradation of isoflavones and color alterations during the drying of okara. *LWT.* (2017) 75:512–9. 10.1016/j.lwt.2016.09.031

[32] Weil M Sing ASC Méot J Boulanger R Bohuon P. Impact of blanching, sweating and drying operations on pungency, aroma and color of *Piper borbonense*. *Food Chem.* (2017) 219:274–81. 10.1016/j.foodchem.2016.09.144 27765227

[33] Horuz E Bozkurt H Karatas H Maskan M. Effects of hybrid (microwave-convectional) and convectional drying on drying kinetics, total phenolics, antioxidant capacity, vitamin C, color and rehydration capacity of sour cherries. *Food Chem.* (2017) 230:295–305. 10.1016/j.foodchem.2017.03.046 28407914

[34] Samoticha J Wojdyło A Lech K. The influence of different the drying methods on chemical composition and antioxidant activity in chokeberries. *LWT.* (2016) 66:484–9. 10.3390/molecules26113274 34071647 PMC8197958

[35] Sablani SS. Drying of fruits and vegetables: retention of nutritional/functional quality. *Drying Technol.* (2006) 24:123–35. 10.1080/07373930600558904

[36] Alanon ME Palomo ES Castro L Vinas MG Perez-Coello MS. Changes produced in the aroma compounds and structural integrity of basil (*Ocimum basilicum* L.) during drying. *J Sci Food Agric.* (2004) 84:2070–6. 10.1002/jsfa.1921

[37] Calín-Sánchez A Kharaghani A Lech K Figiel A Carbonell-Barrachina A Tsotsas E. Drying kinetics and microstructural and sensory properties of black chokeberry (*Aronia melanocarpa*) as affected by drying method. *Food Bioprocess Technol.* (2014) 8:63–74. 10.1007/s11947-014-1383-x

[38] Politowicz J Lech K Lipan L Figiel A Carbonell-Barrachina Á. Volatile composition and sensory profile of shiitake mushrooms as affected by drying method. *J Sci Food Agric.* (2017) 98:1511–21. 10.1002/jsfa.8622 28802017

[39] Nofer J Lech K Sánchez-Rodríguez L Figiel A Szumny A Grubor M Volatile composition and sensory profile of oyster mushroom as affected by drying method. *Drying Technol.* (2018) 36:685–96. 10.1080/07373937.2016.1274903

[40] Ji LL Mu TH Sun HN. Analysis of mass and heat transfer of sweet potato leaves during different drying processes by low field nuclear magnetic resonance. *Food Sci.* (2020) 41:90–6.

[41] Feng D Wang J Ji XJ Min WX Yan WJ. Analysis of volatile organic compounds by HS-GC-IMS in powdered yak milk processed under different sterilization conditions. *J Food Qual.* (2021) 2021:1–10. 10.1155/2021/5536645

[42] Jia W Zhang R Shi L Zhang F Chang J Chu XG. Accurate determination of volatile-flavor components in bosgrunniens milk by high-throughput dynamic headspace gas chromatographic-mass spectrometry. *J Chromatogr A.* (2019) 1603:67–82. 10.1016/j.chroma.2019.06.058 31272730

[43] Chen L Li X Li ZM Deng LG. Analysis of 17 elements in cow, goat, buffalo, yak, and camel milk by inductively coupled plasma mass spectrometry (ICP-MS). *RSC Adv.* (2020) 10:6736–42. 10.1039/d0ra00390e 35493914 PMC9049741

[44] Calín-Sánchez A Carbonell-Barrachina AA. Flavor and aroma analysis as a tool for quality control of foods. *Foods* (2021) 10:224. 10.3390/foods10020224 33499019 PMC7912021

[45] Ahmed M Akter MS Eun JB. Optimisation of drying conditions for the extraction of -carotene, phenolic and ascorbic acid content from yellow-fleshed sweet potato using response surface methodology. *Int J Food Sci Technol.* (2011) 46:1356–62. 10.1111/j.1365-2621.2011.02612.x 20858156

[46] Diaz-Maroto MC Perez-Coello MS Cabezudo MD. Effect of drying method on the volatiles in bay leaf (*Laurus nobilis* L.). *J Agric Food Chem.* (2002) 50:4520–4. 10.1021/jf011573d 12137470

[47] Raksakantong P Siriamornpun S Meeso N. Effect of drying methods on volatile compounds, fatty acids and antioxidant property of Thai kaffir lime (*Citrus hystrix* D.C.). *Int J Food Sci Technol.* (2012) 47:603–12. 10.1111/j.1365-2621.2011.02883.x

[48] Feng D Wang J Ji XX Min WX Yan WJ. HS-GC IMS detection of volatile organic compounds in yak milkpowder processed by different drying methods. *LWT Food Sci Technol.* (2021) 141:110855. 10.1016/j.lwt.2021.110855

[49] Karam MC Petit J Zimmer D Baudelaire Djantou E Scher J. Effects of drying and grinding in production of fruit and vegetable powders: a review. *J Food Eng.* (2016) 188:32–49. 10.1016/j.jfoodeng.2016.05.001

[50] Nijhuis H Torringa H Muresan S Yuksel D Leguijt C Kloek W. Approaches to improving the quality of dried fruit and vegetables. *Trends Food Sci Technol.* (1998) 9:13–20. 10.1016/s0924-2244(97)00007-1

[51] Ulrich D Hoberg E Bittner T Engewald W Meilchen K. Contribution of volatile compounds to the flavor of cooked asparagus. *Eur Food Res Technol.* (2001) 213:200–4. 10.1007/s002170100349

[52] Bearth A Cousin ME Siegrist M. The consumer’s perception of artificial food additives: influences on acceptance, risk and benefit perceptions. *Food Qual Prefer.* (2014) 38:14–23. 10.1016/j.foodqual.2014.05.008

[53] Eiser JR Coulson NS Eiser C. Adolescents’ perceptions of the costs and benefits of food additives and their presence in different foods. *J Risk Res.* (2002) 5:167–76. 10.1080/13669870010004979

[54] Shim SM Seo SH Lee Y Moon G-I Kim M-S Park J-H. Consumers’ knowledge and safety perceptions of food additives: evaluation on the effectiveness of transmitting information on preservatives. *Food Control.* (2011) 22:1054–60. 10.1016/j.foodcont.2011.01.001

[55] Taylor AJ. Physical chemistry of flavor. *Int J Food Sci Technol.* (1998) 33:53–62.

[56] Coumans WJ Kerkhof PJAM Ruin S. Theoretical and practical aspects of aroma retention in spray drying and freeze drying. *Drying Technol.* (1994) 12:99–149. 10.1080/07373939408959951

[57] Reineccius GA. The spray drying of food flavors. *Drying Technol.* (2004) 22:1289–324. 10.1081/drt-120038731

[58] King CJ. Spray drying: retention of volatile compounds revisited. *Drying Technol.* (1995) 13:1221–40. 10.1080/07373939508917018

[59] Pegiou E Mumm R Acharya P de Vos RCH Hall RD. Green and white Asparagus (*Asparagus officinalis*): a source of developmental, chemical and urinary intrigue. *Metabolites.* (2019) 10:17. 10.3390/metabo10010017 PMC7022954 31881716

[60] Rao LJ Singh M Raghavan B Abraham K. Rosemary (*Rosmarinus ocinalis* L.): impact of drying on its flavor quality. *J Food Qual.* (1998) 21:107–15. 10.1111/j.1745-4557.1998.tb00508.x

[61] Politowicz J Lech K Sánchez-Rodríguez L Szumny A Carbonell-Barrachina Á. Volatile composition and sensory profile of *Cantharellus cibarius* Fr. as affected by drying method. *J Sci Food Agric.* (2017) 97:5223–32. 10.1002/jsfa.8406 28466491

[62] Figiel A. Drying kinetics and quality of beetroots dehydrated by combination of convective and vacuum-microwave methods. *J Food Eng.* (2010) 98:461–70. 10.1016/j.jfoodeng.2010.01.029

[63] Nowicka P Wojdyło A Lech K Figiel A. Chemical composition, antioxidant capacity, and sensory quality of dried sour cherry fruits pre-dehydrated in fruit concentrates. *Food Bioprocess Technol.* (2015) 8:2076–95. 10.1007/s11947-015-1561-5

